# Comparative study of immunological biomarkers in the carpet shell clams (*Ruditapes decussatus*) from metal-contaminated sites in the South Lagoon of Tunis (Tunisia)

**DOI:** 10.1007/s11356-022-22902-3

**Published:** 2022-09-14

**Authors:** Chalbia Mansour, María Ángeles Esteban, Omar Rouane Hacene, Dalila Saidane Mosbahi, Francisco Antonio Guardiola

**Affiliations:** 1 Laboratory of Analysis, Treatment and Valorization of Pollutants of the Environment and Products, Faculty of Pharmacy, University of Monastir, Monastir, Tunisia; 2 Immunobiology for Aquaculture Group, Department of Cell Biology and Histology, Faculty of Biology, Regional Campus of International Excellence “Campus Mare Nostrum”, University of Murcia, 30100 Murcia, Spain; 3 Laboratoire Réseau de Surveillance Environnementale (LRSE), Department of Biology, University of Oran, 1 Ahmed Ben Bella, BP 1524 El M’naouer, 31000 Oran, Algeria

**Keywords:** Biomarkers, Biomonitoring, Innate immunity, Seasonality, Metal contamination, South Lagoon of Tunis, Carpet shell clam (*Ruditapes decussatus*)

## Abstract

The South Lagoon of Tunis (Tunisia) is a Mediterranean lagoon adversely affected by industrial contaminants, harbour activity and untreated urban sewage. In this lagoon, the clam *Ruditapes decussatus* has been widely used as a biomonitor of seawater pollution through measurements of parameters related to oxidative stress and neurotoxicity. However, few studies have considered parameters of the immune system of this species in the South Lagoon of Tunis. Therefore, the aim of the present work was to evaluate several immune-related parameters in the cell-free haemolymph of carpet shell clams sampled during August and February from three polluted sites in the South Lagoon of Tunis (S1, S2 and S3) and one less polluted site as a reference site (RS) in order to identify suitable biomarkers for environmental quality assessments of this ecosystem. Concerning the immune-related parameters, seasonal factors modulated phenoloxidase, lysozyme, protease and esterase activity, with lower values measured for samples collected in August than for samples collected in February. In fact, bactericidal activity against two of the pathogenic bacteria tested and the activity of most immune-related enzymes were reduced in the cell-free haemolymph of clams collected from the most sampling sites in August compared to February one. In addition, values of abiotic parameters (temperature, salinity and pH) and metal (cadmium, copper, iron, lead and zinc) concentrations in the clams’ soft tissues, previously obtained and published by the authors, as well as the values of immune-related parameters were integrated using principal component analyses. Results indicated that the values of all measured immune-related parameters were negatively correlated with the temperature values and the variations most of these parameters highlighted that the chemical industrial area (S3) was the most impacted location within the South Lagoon of Tunis. The present study illustrates that the immune-related parameters measured in carpet shell clam cell-free haemolymph represent suitable biomarkers for environmental quality assessments because they provide effective seasonal and spatial discrimination.

## Introduction

Marine ecosystems are subject to much environmental concern owing to an accelerated increase in highly persistent pollutant levels of polycyclic aromatic hydrocarbons (PAHs), heavy metals, pesticides and microplastics, amongst other substances (Capó et al. 2015; Breitwieser et al. 2016; Hong et al. 2016; Gola et al. 2021; Shah 2021; Zaynab et al. 2021; Zhang et al. 2021). The release of such compounds into the environment can have deleterious effects on aquatic organisms. For this reason, several bivalve species, such as mussels, oysters and clams, are used worldwide as sentinels in pollution monitoring and are considered useful biomonitors due to their sessile nature, filter-feeding behaviour and high bioaccumulation ability (Sandrini-Neto et al. 2016; Yap et al. 2021).

Chemical analyses of toxic compounds in bivalve tissues can provide useful information, but they do not necessarily indicate these contaminants’ toxicological effect on living organisms (Solé 2000). Biochemical, cellular and physiological analyses detect exposure to several pollutants in a rapid and precise approach, allowing earlier identification of changes in aquatic organisms before adverse effects reach higher organisation levels and, ultimately, the ecosystem (Monserrat et al. 2003). In the last 20 years, to predict the potential risk of marine contamination, many researchers have employed a suite of biomarkers. For example, acetylcholinesterase activity in bivalves has been widely used as a specific biomarker to indicate the presence of neurotoxic compounds, such as organophosphorus and carbamates (Moncaleano-Niño et al. 2022). Similarly, metallothione measurements in bivalves are commonly used as a specific biomarker of metallic trace element exposure (Moncaleano-Niño et al. 2022). In contrast, measures of antioxidant responses and oxidative stress damage (e.g., glutathione-S-transferase, catalase, lipid peroxidation and protein oxidation) are considered non-specific biomarkers (Mansour et al. 2020). Concerning to an immunological point of view, several studies have reported different disorders induced by chemical contaminants in several bivalve species (Zha et al. 2019; Sun et al. 2020; Tang et al. 2020). In this context, researchers have proposed that immunological biomarkers are sensitive tools in eco-immunology studies for detecting signs of impaired bivalve health (Auffret et al. 2006; Cotou et al. 2013; Matozzo et al. 2013).

To use biomarkers in monitoring programmes, one must take biotic and abiotic factors into consideration. A wide variety of studies have demonstrated that several abiotic and biotic factors—such as temperature, salinity, food availability and reproduction process—have the potential to modulate changes in biomarkers caused by chemical stress in bivalve molluscs (Pokhrel et al. 2021). Therefore, an integrated chemical-biological approach must be taken into account in ecotoxicological studies assessing environmental quality.

The study area of the present work, the South Lagoon of Tunis (Tunisia), is a Mediterranean lagoon located in the southwest of the Gulf of Tunis and connected to the sea through the Rades Canal (Jouini et al. 2005). The lagoon is adversely affected by industrial contaminants from the industrial zone, substantial harbour activity and untreated urban sewage from Tunis and its southern suburbs (Jouini et al. 2005). In fact, high levels of PAHs (Mzoughi and Chouba 2011; Chalghmi et al. 2020; Mansour et al. 2021) and heavy metals—such as mercury (Hg), zinc (Zn), cadmium (Cd), lead (Pb) and nickel (Ni) (Chalghmi et al. 2016; Mansour et al. 2020)—have recently been found in lagoon sediments. The carpet shell clam (*Ruditapes decussatus*), which is widely distributed in European and Mediterranean coastal waters and of great ecological and economic importance (Cravo et al. 2012), has been broadly used as a biomonitor of seawater pollution in the South Lagoon of Tunis. However, existing studies have paid special attention to measuring parameters related to oxidative stress, neurotoxicity and histopathological alterations, mainly in the gills and the digestive gland (Bejaoui et al. 2018, 2020), whilst immune-related parameters analysed in cell-free haemolymph have received little to no attention. In light of these considerations, the aim of the present study was to evaluate the alterations in a battery of immunological biomarker responses in the cell-free haemolymph of carpet shell clams collected from the South Lagoon of Tunis (influenced by anthropogenic impact) to identify immune-related parameters that could potentially be employed as biomarkers of environmental pollution. The selected biomarkers could provide a valuable information of the environmental quality of seawater in monitoring programmes, an approach that could prove very useful in ecosystems that are characterised by complex mixtures of contaminants.

## Material and methods

### Study area, sampling sites and sample collection

**Fig. 1:**
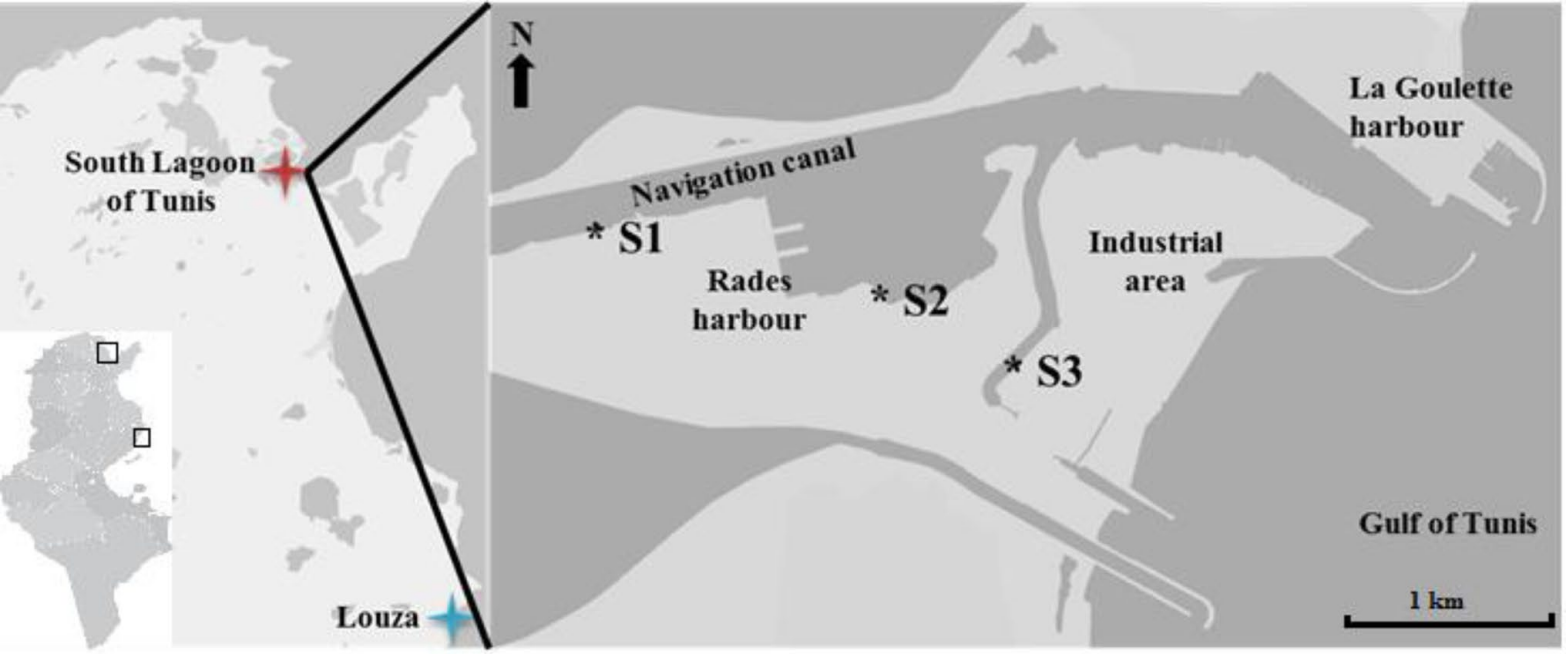
Map of the study area and location of the sampling points (S1, S2 and S3) in the South Lagoon of Tunis and the reference site (RS) in Louza

European carpet shell clams were collected from three different areas (S1, S2 and S3) in the South Lagoon of Tunis (Fig. 1) located near different contamination sources. Site S1 was located in the middle of the navigation canal that connects the harbour of La Goulette to Tunis and Rades harbours (10°14′41.6″W, 36°48′15.3″N). Site S2 was located very close to Rades harbour (10°16′19.1″W, 36°48′12.4″N) which is the largest commercial harbour in Tunis (more polluted than S1) which has the most intense commercial transport activities. Site S3 was located closer to the petrochemical industrial area and the Rades power station which is the largest power station in Tunisia (10°16′53.1″W, 36°47′59.6″N). These industries pump its sewage into the lagoon. In fact, high levels of PAHs (Mzoughi and Chouba 2011; Chalghmi et al. 2020; Mansour et al. 2021) and heavy metals—such as mercury, zinc (Zn), cadmium (Cd), lead (Pb) and nickel (Chalghmi et al. 2016; Mansour et al. 2020)—have recently been found in lagoon sediments and clams. Control clams were collected from a coastal location at Louza as reference site (RS) (35°02′00.1″N 11°00′66.3″E), which has been considered a less polluted site in monitoring programmes along the Tunisian coasts (Banni et al. 2009) (Fig. 1).

Clams were sampled by hand (100 ± 20 SD per site and per month), rather than being harvested through automated processes, during August (August 2015) and February (February 2016). The clams were then put in clean plastic bags and transported to the laboratory at 4 °C. Upon arrival to the laboratory, clams were transferred to aquaria filled with aerated seawater from each sampling sites (24 °C in August and 13 °C in February) for 24 h (holding phase to minimise the effect of sampling and transport on the immune parameters) and the length of the clam’s shell was measured (data shown in Mansour et al. 2020). The next day, samples of 1 mL of haemolymph were collected from the anterior adductor muscle of each clam with a 2-mL plastic syringe. Then, all individual sample were centrifuged (780 × *g*, 10 min at 4 °C) and the supernatants, corresponding to cell-free haemolymph, were collected and stored at − 20 °C until analysis. Each biochemical determination was carried out on 20 specimens and each measurement was performed in triplicate.

### Physical–chemical characterisation

The physical–chemical characterisation of the seawater (salinity, temperature and pH) was measured during the clam sampling at the four selected sites in order to provide information of water quality. Briefly, the measurement of seawater temperature was carried out in situ whilst the salinity and the pH values were determined in the laboratory in 1-L samples of seawater collected in glace bottles. All the parameters were measured in triplicate. The values of these abiotic parameters have been published recently (Mansour et al. 2020) and are shown in Table 1 of the present study.

**Table 1:** Values of salinity (practical salinity units, psu), temperature (°C) and pH measured in seawater from three lagoon sites (S1, S2 and S3) and one as reference site (RS) in August and February. Data represent the mean ± SEM. Asterisks indicate significant differences between the two months (August and February) in each sampling point, whilst lower- and upper-case letters denote significant variations between sampling points in August and February months, respectively (two-way ANOVA, *p* < 0.05). All the parameters were measured in triplicate in order to calculate the mean and SEM; however, in the case of salinity values, the three values were equal for each sampling site

Parameters	Season	Locations sampled			
		RS	S1	S2	S3
Salinity (psu)	August	39.47 ± 0.00	40.90 ± 0.00	40.70 ± 0.00	40.70 ± 0.00
	February	39.58 ± 0.00	34.90 ± 0.00	35.05 ± 0.00	35.09 ± 0.00
Temperature (°C)	August	27.85 ± 0.27a*	23.93 ± 0.38b*	23.45 ± 0.37b*	24.05 ± 0.36b*
	February	14.28 ± 0.07	12.67 ± 0.16	12.6 ± 0.15	12.65 ± 0.17
pH	August	8.27 ± 0.02a	8.12 ± 0.05b	8.21 ± 0.01ab	7.85 ± 0.07c*
	February	8.14 ± 0.02AB	8.11 ± 0.03AB	8.24 ± 0.02A	8.02 ± 0.01B

### Metal analysis

The concentrations of cadmium (Cd), copper (Cu), iron (Fe), lead (Pb) and zinc (Zn) were measured in the whole soft tissues of clams (Mansour et al. 2020). Briefly, 250 mg of dry weight were digested in 1 mL of nitric acid (1 N) at 95 °C for 1 h. The liquid underwent fivefold dilution with ultrapure water. After that, the metal contents in acid solutions were determined by using a flame atomic absorption spectrophotometer equipped with a graphite furnace (PerkinElmer AAnalyst-100 version 1.10). Quality assurance and quality control were assessed by processing blank samples and reference standard material (Mussel Tissue Standard Reference Material SRM 2976, National Institute of Standards and Technology). All metal concentrations were reported in micrograms per gramme of sample dry weight. The values of metal concentrations have been published recently (Mansour et al. 2020) and are shown in Table 2 of the present study.

**Table 2:** Concentrations (μg g dry weight^−1^) of cadmium (Cd), copper (Cu), iron (Fe), lead (Pb) and zinc (Zn) in the soft tissues of carpet shell clam (*Ruditapes decussatus*) collected from three lagoon sites (S1, S2 and S3) and one as reference site (RS) in August and February months. Data represent the mean ± SEM. Different letters denote significant variations between sampling sites (ANOVA, *p* < 0.05)

Metal concentrations (μg g dry weight^−1^)	Locations sampled			
	RS	S1	S2	S3
Cadmium (Cd)	0.43 ± 0.05a	0.67 ± 0.06b	0.51 ± 0.07ab	0.47 ± 0.06a
Copper (Cu)	4.08 ± 0.39	3.70 ± 0.51	3.99 ± 0.46	3.51 ± 0.45
Iron (Fe)	68.62 ± 3.26a	118.77 ± 4.68b	78.07 ± 5.55ac	98.97 ± 11.46bc
Lead (Pb)	0.41 ± 0.02a	0.94 ± 0.03b	0.75 ± 0.04ab	0.61 ± 0.03a
Zinc (Zn)	21.61 ± 0.85a	53.74 ± 2.23b	51.11 ± 3.86b	57.32 ± 2.90b

### Protein determination

The total protein concentration in cell-free haemolymph samples was determined using the method described by Bradford (1976). Serial dilutions of bovine serum albumin (Sigma-Aldrich, St. Louis, MO, USA) were used as a standard. Plates were read at 550 nm in a plate reader (Spectro UV–Vis Double BEAM PC, LABOMED, INC). The total protein concentration present in each sample was expressed as milligrammes per millilitre.

### Enzymatic activities

#### Phenoloxidase activity

Phenoloxidase activity was determined according to the method described by Asokan et al. (1997). Briefly, 50 µL of cell-free haemolymph was incubated with the same volume of SDS (sodium dodecyl sulphate, 1 mg mL^−1^, Sigma) for 5 min at room temperature. Then, 50 µL of l-DOPA (3,4-dihydroxyphénylalanine, Sigma) (3 mg mL^−1^ in 0.5 M HCl containing 10 mM CaCl_2_) was added as substrate, and the optical density was recorded at 490 nm every minute for 15 min in a plate reader (FLUOstar OPTIMA). Enzyme activity was expressed as units, where one unit represents the change in absorbance per minute per milligramme of protein.

#### Lysozyme activity

The turbidimetric method described by Parry et al. (1965) was adopted to determine the lysozyme activity. In 96-well flat-bottomed plates, a volume of 100 µL of cell-free haemolymph was mixed the same volume of freeze-dried *Micrococcus lysodeikticus* (0.3 mg mL^−1^, Sigma) as lysozyme substrate. Then, the reduction in absorbance at 450 nm was measured over the course of 15 min at 22 °C in a plate reader (FLUOstar OPTIMA). One unit of lysozyme activity was defined as a reduction in absorbance of 0.001 min^−1^. The units of lysozyme present in cell-free haemolymph were obtained from a standard curve made with hen egg white lysozyme (HEWL, Sigma) and the enzymatic activity was expressed as microgrammes per milligramme of cell-free haemolymph proteins.

#### Alkaline phosphatase activity

Alkaline phosphatase activity in cell-free haemolymph samples was measured according to the method of Guardiola et al. (2014) with slight modifications. Aliquots of 100 µL of cell-free haemolymph were mixed with the same volume of 4 mM p-nitrophenyl liquid phosphate (Sigma) in 100 mM ammonium bicarbonate buffer containing 1 mM MgCl_2_ (pH 7.8, 30 °C). The OD was continuously measured at 405 nm at 1-min intervals over the course of 1 h in a plate reader (FLUOstar OPTIMA). One unit of activity was defined as the amount of enzyme required to release 1 µmol of p-nitrophenol phosphate product in 1 min and the activity was expressed as U mg^−1^ cell-free haemolymph proteins.

#### Esterase activity

Esterase activity in cell-free haemolymph samples was measured by mixing an equal volume of samples with 0.4 mM p-nitrophenylmyristate as substrate, in 100 mM ammonium bicarbonate buffer containing 0.5% Triton X-100 (pH 7.8, 30 °C) as described by Guardiola et al. (2014) with slight modifications. The OD was continuously measured at 1-min intervals over 1 h at 405 nm in a plate reader (FLUOstar OPTIMA). Standard samples without cell-free haemolymph were used as blanks. The esterase activity was expressed as units per milligramme of cell-free haemolymph proteins where one unit of activity was defined as the amount of enzyme required to release 1 µmol of p-nitrophenyl myristate (Sigma) product in 1 min.

#### Peroxidase activity

The peroxidase activity was quantified as described by Quade and Roth (1997). Briefly, a volume of 10 μL of cell-free haemolymph was mixed with 40 μL of Hank’s buffer without Ca^+2^ or Mg^+2^ in a 96-well plate. Then, 50 μL of 20 mM TMB (3,3′,5,5′-tetramethylbenzidine hydrochloride, Sigma) and 5 mM of H_2_O_2_ were added. After 2 min, 50 μL of sulphuric acid (2 M) was added to stop the reaction and the optic density was measured at 450 nm in a plate reader (FLUOstar OPTIMA). The enzymatic activity was expressed as units per milligramme of protein, where one unit represents the amount producing a change in absorbance.

#### Protease activity

The protease activity was measured according to the colorimetric method described by Guardiola et al. (2018) with slight modifications. A volume of 10 μL of cell-free haemolymph was incubated with 100 μL of ammonium bicarbonate buffer (100 mM) and 125 μL of azocasein (2%) for 24 h at room temperature. Then, 250 μL of 10% trichloroacetic acid (TCA) was added to stop the reaction. After centrifugation (10,000 × *g*, 10 min), 100 μL of supernatant was transferred in triplicate to a 96-well plate containing 100 μL of NaOH (1 N) per well and the optical density was measured at 405 nm in a plate reader (FLUOstar OPTIMA). The cell-free haemolymph was replaced by trypsin solution (5 mg mL^−1^) as positive control (100% protease activity) and by buffer as negative control (0% protease activity).

#### Antiprotease activity

The method of Hanif et al. (2004) modified by Bahi et al. (2017) was used for the measurement of the anti-protease activity. The assay consists of estimating the cell-free haemolymph capacity to inhibit trypsin activity. A volume of 10 μL of cell-free haemolymph was incubated for 10 min at 25 °C with 10 μL of a trypsin solution (5 mg mL^−1^). After that, 100 μL of 100 mM ammonium bicarbonate buffer and 125 μL of 0.7% azocasein were added. Then, the samples were incubated for 2 h at 30 °C. A second incubation for 30 min at 30 °C was carried out after adding 250 μL of TCA at 4.6%. The mixture was then centrifuged (10,000 × *g*, 10 min) and the supernatant was transferred in triplicate on a 96-well plate containing 100 μL of 0.5 N NaOH. The buffer replaced the cell-free haemolymph for positive control (100% protease and 0% antiprotease activity), and trypsin for negative control (0% protease and 100% antiprotease activity). OD was recorded at 450 nm in a plate reader (FLUOstar OPTIMA) and the percentage inhibition of trypsin activity was calculated.

### Bactericidal activity

The Sunyer and Tort (1995) method with some modifications was used to determine the bactericidal activity in the cell-free haemolymph. Three pathogenic bacteria (*Vibrio anguillarum*, *Photobacterium damselae* subsp. *piscicida* and *Escherichia coli*) were used to determine the bactericidal activity. The bacteria were cultured on Tryptone Soy Broth (Sigma) at 25 °C. Aliquots of 100 μL of bacterial suspension (10^6^ CFU mL^−1^ bacteria) were placed in 96-well flat-bottomed plates and incubated for 5 h at 25 °C with equal volumes of cell-free haemolymph samples. A volume of 25 μL of MTT (3-(4,5 dimethyl-2-yl)-2,5-diphenyl tetrazolium bromide, 1 mg mL^−1^) was added to each well. Thereafter, the plates were incubated for 10 min at 25 °C and then centrifuged (2,000 × *g* for 10 min). The precipitate was dissolved in 200 μL of DMSO (dimethyl sulfoxide) and transferred to a flat-bottom 96-well plate. The absorbance was recorded at 570 and 690 nm (final Abs = Abs_570_ − Abs_690_). Samples without bacteria were used as blanks (negative control). The bactericidal activity was determined according to the following formulas:

$$\begin{array}{c}-\%\;\mathrm{viable}\;\mathrm{bacteria}=\left(\mathrm{Sample}\;\mathrm{Abs}\times100\right)/\mathrm{Abs}.\;\mathrm{of}\;\mathrm{the}\;\mathrm{reference}\;\mathrm{sample}\\-\%\;\mathrm{noviable}\;\mathrm{bacteria}\;\left(\mathrm{bactericidal}\;\mathrm{activity}\right)=100\%\;\mathrm{viable}\;\mathrm{bacteria}\end{array}$$

### Statistical analysis

All measurements were performed on three replicates. The results are expressed as mean ± standard error of the mean (SEM). Data were statistically analysed using two-way analysis of variance (ANOVA) followed by Tukey test to determine differences between the values obtained in the cell-free haemolymph of clams from sampling sites and amongst seasonal points (August and February), respectively. Non-normally distributed data were log-transformed prior to analysis, and a non-parametric Kruskal–Wallis test, followed by a multiple comparison test, was used when data did not meet parametric assumptions. Statistical analyses were conducted using SPSS 19 and differences were considered statistically significant when *p* < 0.05.

Pearson’s correlation analysis was performed to identify pairwise associations between immune-related parameters as biomarkers, metal concentrations (Cd, Cu, Fe, Pb and Zn) and abiotic parameters (salinity, temperature and pH) using the R Core Team (2020). A correlation coefficient higher than 0.7 was considered significant at *p* < 0.05. Additionally, principal component analysis (PCA) was also applied to the whole data set, including biomarkers (immune-related parameters), environmental parameters (abiotic parameters (salinity, temperature and pH) and metal concentrations (Cd, Cu, Fe, Pb and Zn) in the whole soft tissues of clams) and sampling sites to evaluate the relationships between variables using the software STATISTICA (Statsoft STATISTICA version 6.1.478.0).

## Results

### Enzymatic activity measured in the cell-free haemolymph of clams

**Fig. 2:**
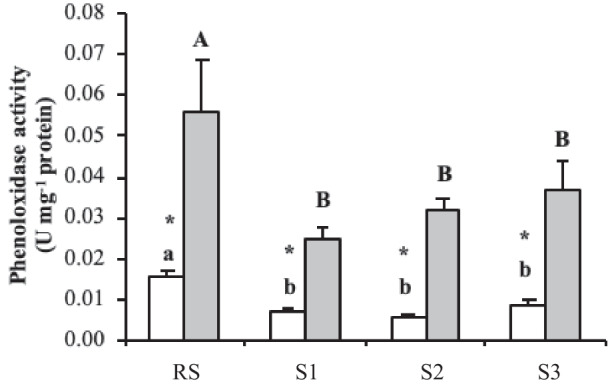
Phenoloxidase activity (U mg.^−1^ protein) in the cell-free haemolymph of carpet shell clams (*Ruditapes decussatus*) collected from three lagoon (S1, S2 and S3) and the RS (reference site) during August (white bars) and February (grey bars). The bars represent the mean ± SEM. Asterisks indicate significant differences between the two seasonal points (August and February) for each sampling site, whilst lowercase and uppercase letters denote significant variations between sampling sites for August and February, respectively (two-way ANOVA, *p* < 0.05)

Phenoloxidase activity was overall lower in the cell-free haemolymph of clams sampled from the three contaminated lagoon sites (S1, S2 and S3) than in the cell-free haemolymph of clams from the RS for August and February (Fig. 2). Interestingly, a drastic reduction in phenoloxidase activity was observed in clams collected during August as compared to those collected during February for all sampling points.

**Fig. 3:**
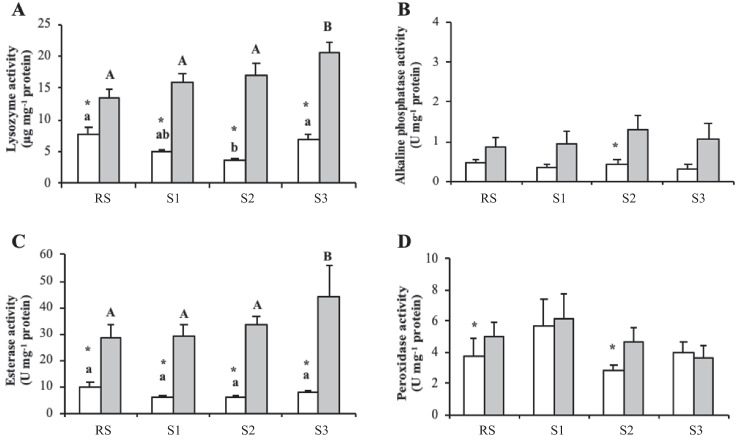
Lysozyme (µg mg^−1^ protein) (**A**), alkaline phosphatase (U mg^−1^ protein) (**B**), esterase (U mg^−1^ protein) (**C**) and peroxidase (U mg.^−1^ protein) (**D**) activity in the cell-free haemolymph of carpet shell clams (*Ruditapes decussatus*) collected from three lagoon (S1, S2 and S3) and the RS (reference site) during August (white bars) and February (grey bars). The bars represent the mean ± SEM. Asterisks indicate significant differences between the two seasonal points (August and February) for each sampling site, whilst lowercase and uppercase letters denote significant variations between sampling sites for August and February, respectively (two-way ANOVA, *p* < 0.05)

Regarding lysozyme and esterase activity, levels of both enzymes were lower in the cell-free haemolymph of clams collected in August compared to those collected in February for all experimental sampling sites (including SR) (Fig. 3A, C). Registered activity for both enzymes was the highest in the cell-free haemolymph of clams from S3 in February compared to the other sampling points. In addition, a reduction in lysozyme activity was recorded in the cell-free haemolymph of clams collected from S2, as compared to the RS and S3, for the August (Fig. 3A). Regarding alkaline phosphatase and peroxidase activity, no variations were observed in the cell-free haemolymph of clams from the four sampling sites for August and February (Fig. 3B, D). However, levels of both enzymes were higher in the cell-free haemolymph of clams collected in February from S2 compared to August, whereas peroxidase activity was also higher in clams collected in February than in August for the RS.

**Fig. 4:**
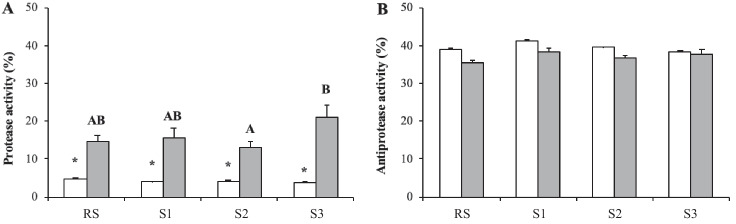
Protease (%) (**A**) and antiprotease (%) (**B**) activity in the cell-free haemolymph of carpet shell clams (*Ruditapes decussatus*) collected from three lagoon (S1, S2 and S3) and the RS (reference site) during August (white bars) and February (grey bars). The bars represent the mean ± SEM. Asterisks indicate significant differences between the two seasonal points (August and February) for each sampling site, whilst uppercase letters denote significant variations between sampling sites (two-way ANOVA, *p* < 0.05)

In terms of protease activity, no spatial variations were observed in the cell-free haemolymph of clams sampled in August, whilst the protease activity levels in February were higher in clams collected from S3 than in clams from S2 (Fig. 4A). Interestingly, recorded protease activity was higher in February than in August, regardless of the sampling point. In contrast, antiprotease activity measured in the cell-free haemolymph of clams exhibited no significant variations between either sampling sites or the two studied months (August and February) (Fig. 4B).

### Bactericidal activity

**Fig. 5:**
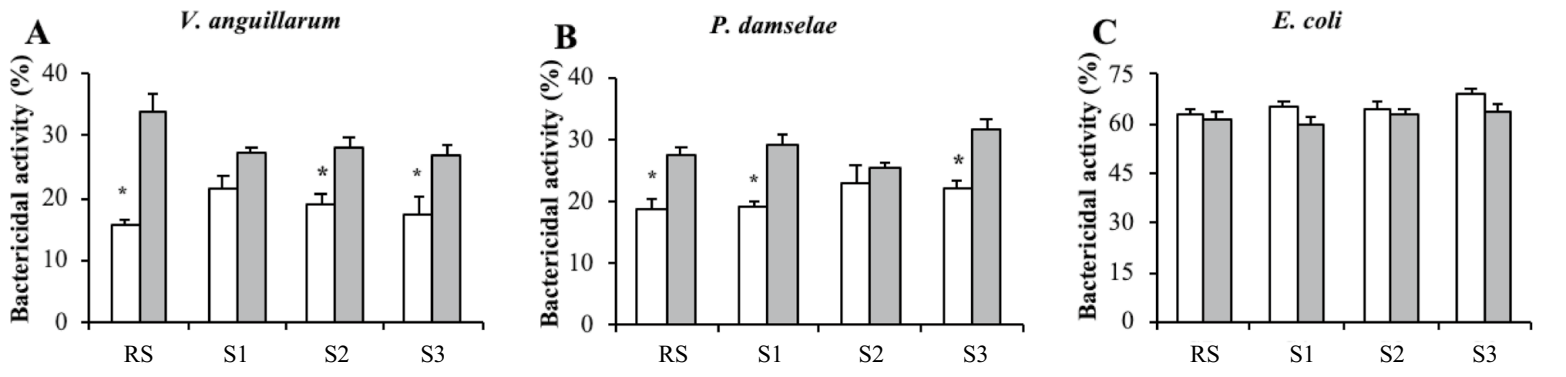
Bactericidal activity (%) against *Vibrio anguillarum* (**A**), *Photobacterium damselae* subsp. *piscicida* (**B**) and *Escherichia coli* (**C**) in the cell-free haemolymph of carpet shell clams (*Ruditapes decussatus*) collected from three lagoon (S1, S2 and S3) and the RS (reference site) during August (white bars) and February (grey bars). The bars represent the mean ± SEM. Asterisks indicate significant differences between the two seasonal points (August and February) for each sampling site (two-way ANOVA, *p* < 0.05)

Results on the cell-free haemolymph bactericidal activity of the clams against the three bacteria tested (*V. anguillarum*, *P. damselae* and *E. coli*) did not reveal variations amongst the sampling sites for August and February (Fig. 5). However, cell-free haemolymph bactericidal activity was higher in clams collected in February compared to those sampled in August. More specifically, bactericidal activity was higher in the case of *V. anguillarum* in the cell-free haemolymph of clams collected from the RS, S2 and S3 (Fig. 5A) and in the case of *P. damselae* in the cell-free haemolymph of clams from the RS, S1 and S3 (Fig. 5B).

### Principal component analysis and Pearson’s correlation results

**Fig. 6:**
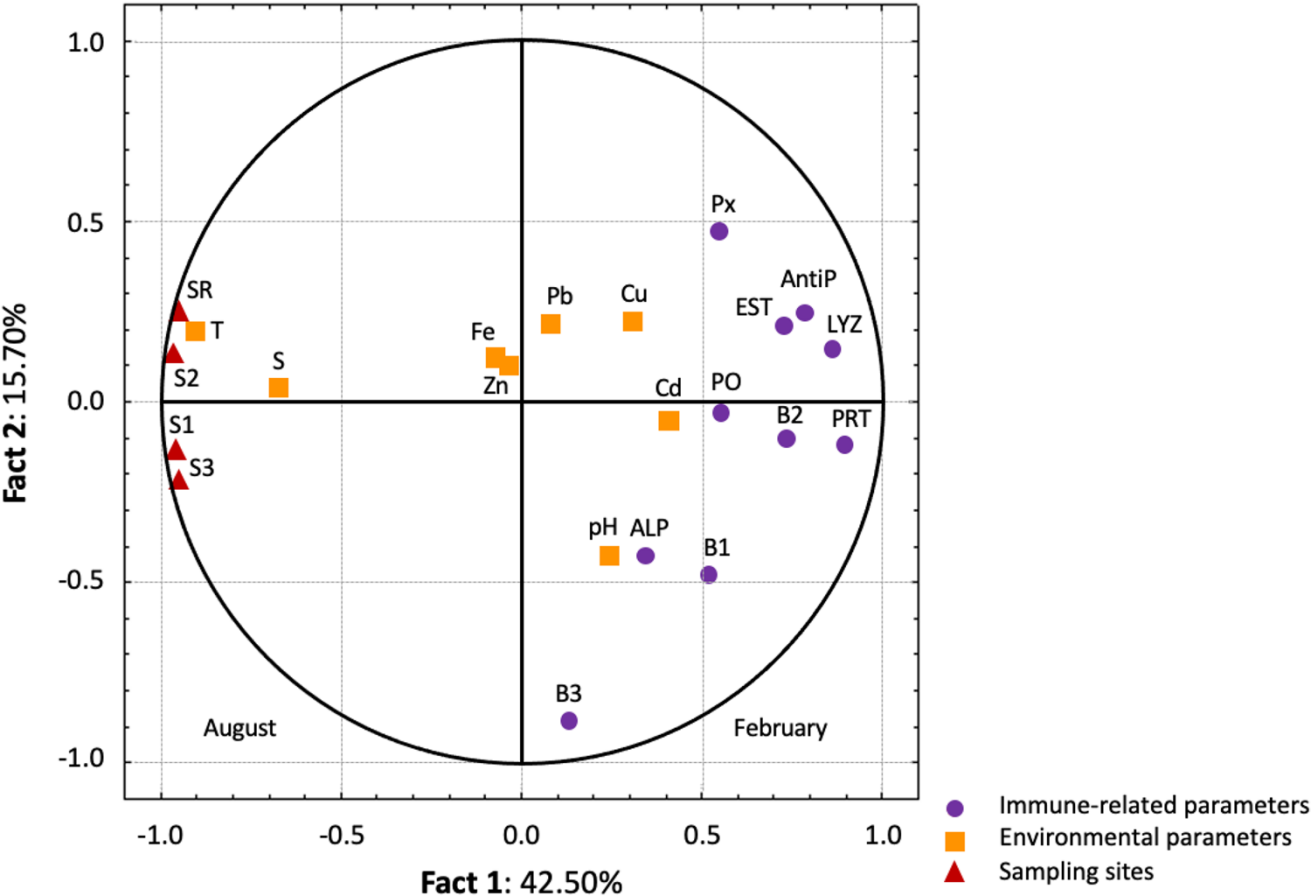
Principal component analysis of the two main factors (F1 *vs*. F2) produced by the environmental parameters—salinity, temperature, pH, heavy metal concentration (Cd, Cu, Fe, Pb and Zn) measured in whole soft tissues—and immune-related parameters measured in the cell-free haemolymph of carpet shell clams (*Ruditapes decussatus*) collected from three lagoon (S1, S2 and S3) and the RS (reference site) during August and February. Legend: PO, phenoloxidase; Px, peroxidase; PRT, protease; AntiP, antiprotease; EST, esterase; ALP, alkaline phosphatase; LYZ, lysozyme; B1, bactericidal activity against *V. anguillarum*; B2, bactericidal activity against *P. damselae*; B3, bactericidal activity against *E. coli*; S, salinity; T, temperature; Cd, cadmium; Cu, copper; Fe, iron; Zn: zinc; and Pb, lead

The PCA performed on the biomarker, environmental parameters and sampling site data (24 variables) identified two main factors, which explained 58.20% of the total variance (Fig. 6). Factor 1 showed a clear seasonal separation in the *x*-axis that explained 42.50% of the total variance in the August, whilst a general increase in biomarker responses was recorded for February. Factor 2 (*y*-axis) explained 15.70% of the total variance. This axis confirmed that the S3 location was the most impacted South Lagoon of Tunis site. The high correlation loadings in the first component indicated that the values of all measured immune-related parameters were negatively correlated with the temperature values. In the present study, correlation between the different variables with principal component axes, factor 1 and factor 2, is shown in Table 3 including all sampling sites during August and February. Coefficients higher than 0.5 indicate a good representation of the variables with factor 1 and factor 2. In this study, factor 1 was positively correlated with phenoloxidase, protease, antiprotease, esterase, lysozyme and bactericidal activity against *P. damselae* activity but negatively correlated with salinity and temperature. On the other hand, factor 2 was positively correlated with bactericidal activity against *V. anguillarum* and Cd concentration but negatively correlated with Fe and Zn presence in clam tissues.

**Table 3:** Principal component analyses (PCA): correlations between variables and principal components including all sampling sites in August and February months. Values in bold indicate significant correlation between the variable and the principal component

Variables	Factor 1	Factor 2
PO	**0.508**	0.348
Px	0.493	0.064
PRT	**0.864**	0.123
AntiP	**0.783**	− 0.093
EST	**0.677**	− 0.006
ALP	0.423	− 0.110
LYZ	**0.891**	− 0.166
B1	0.495	**0.514**
B2	**0.707**	− 0.034
B3	0.187	0.249
S	**-0.785**	0.499
T	− **0.957**	− 0.011
pH	0.386	0.011
Cd	0.348	**0.798**
Cu	0.412	− 0.472
Fe	− 0.053	− **0.691**
Zn	0.015	− **0.795**
Pb	0.135	− 0.387

*PO* phenoloxidase, *Px* peroxidase, *PRT* protease, *AntiP* antiprotease, *EST* esterase, *ALP* alkaline phosphatase, *LYZ* lysozyme, *B1* bactericidal activity against *V. anguillarum*, *B2* bactericidal activity against *P. damselae*, *B3* bactericidal activity against *E. coli*, *S* salinity, *T* temperature, *Cd* cadmium, *Cu* copper, *Fe* iron, *Zn*: zinc, *Pb* lead

**Fig. 7:**
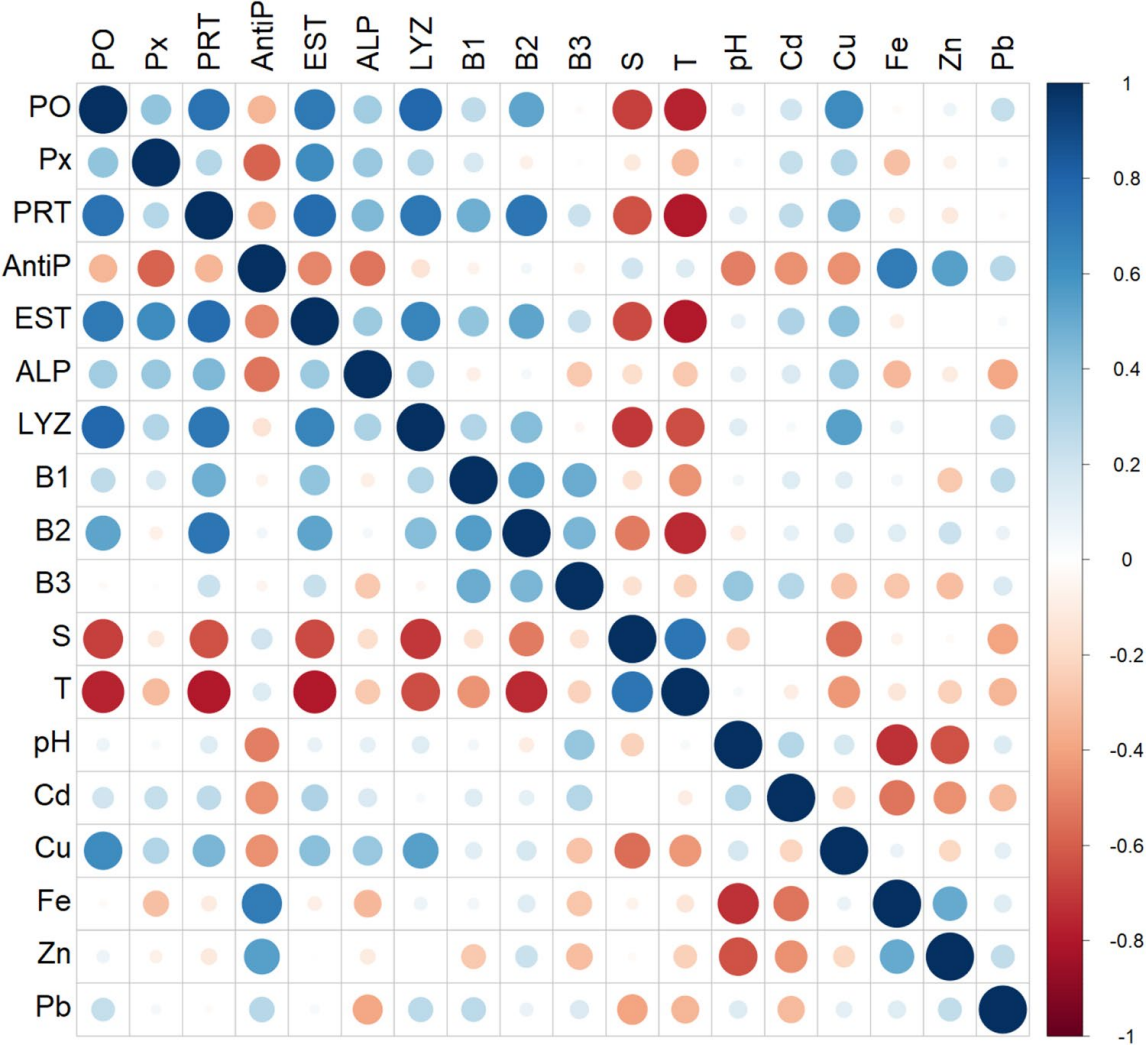
Correlation matrix of environmental environmental parameters—salinity (S), temperature (T), pH, heavy metal concentration (Cd, Cu, Fe, Pb and Zn) measured in whole soft tissues—and immune-related parameters measured in the cell-free haemolymph of carpet shell clams (*Ruditapes decussatus*) collected from three lagoon (S1, S2 and S3) and the RS (reference site) during August and February. Legend: PO, phenoloxidase; Px, peroxidase; PRT, protease; AntiP, antiprotease; EST, esterase; ALP, alkaline phosphatase; LYZ, lysozyme; B1, bactericidal activity against *V. anguillarum*; B2, bactericidal activity against *P. damselae*; B3, bactericidal activity against *E. coli*; S, salinity; T, temperature; Cd, cadmium; Cu, copper; Fe, iron; Zn, zinc; and Pb, lead. Darker blue values are strong positive correlations and red purple values are strong negative correlations, whilst dimmer blue and red indicate weaker correlations

Regarding Pearson’s correlation, the coefficients between the immune-related parameters studied as biomarkers (phenoloxidase, lysozyme, alkaline phosphatase, esterase, peroxidase, protease, antiprotease and bactericidal activity), contaminants and environmental parameters are shown in Table 4 and Fig. 7 for all sampling sites and August and February. The results confirmed that phenoloxidase activity was positively correlated with protease (*r* = 0.7323), esterase (*r* = 0.7702) and lysozyme (*r* = 0.7968) activity. Similarly, protease activity was positively correlated with esterase (*r* = 0.7991), lysozyme (*r* = 0.7674) and bactericidal activity against *P. damselae* (*r* = 0.7761), whereas esterase activity was positively correlated with lysozyme activity (*r* = 0.7218). Contrarily, protease activity was negatively correlated with salinity (*r* =  − 0.7064) and temperature (*r* =  − 0.7216). Similarly, esterase and lysozyme activity were negatively correlated with temperature (*r* =  − 0.7534) and salinity (*r* =  − 0.8135), respectively. A negative correlation was detected between bactericidal activity against *P. damselae* and temperature (*r* =  − 0.7621).

**Table 4:** Pearson’s correlation coefficients (*r*) of the immune-related parameters studied, considering the sampling sites (three lagoon sites (S1, S2 and S3) and one as reference site (RS)) in August and February months. A correlation coefficient higher than 0.7 was considered significant at *p* < 0.05 (values in bold)

Parameters	PO	Px	PRT	AntiP	EST	ALP	LYZ	B1	B2	B3	S	T	pH	Cd	Cu	Fe	Zn	Pb
PO	1																	
Px	0.2924	1																
PRT	**0.7323**	0.3564	1															
AntiP	− 0.2665	− 0.3105	− 0.1804	1														
EST	**0.7702**	0.5734	**0.7991**	− 0.2498	1													
ALP	0.1563	0.2941	0.5210	− 0.2584	0.3421	1												
LYZ	**0.7968**	0.4274	**0.7674**	− 0.1154	**0.7218**	0.4977	1											
B1	0.1927	0.2497	0.4174	− 0.0442	0.1934	− 0.0514	0.3991	1										
B2	0.4612	− 0.0769	**0.7761**	0.08645	0.6085	0.2227	0.5359	0.5460	1									
B3	− 0.0115	− 0.0271	0.1802	− 0.0623	0.1149	− 0.2407	− 0.0060	0.5181	0.3961	1								
S	− 0.6810	− 0.1934	− **0.7064**	0.0832	− 0.6978	− 0.4040	− **0.8135**	− 0.2243	− 0.6287	− 0.0586	1							
T	− 0.6088	− 0.3802	− **0.7216**	0.0521	− **0.7534**	− 0.4137	− 0.6647	− 0.5918	− **0.7621**	− 0.1803	0.6844	1						
pH	0.0219	− 0.0168	0.0611	− 0.4384	0.0331	0.1570	0.1162	0.0769	− 0.0346	0.4179	− 0.1506	− 0.0255	1					
Cd	0.1113	0.2644	0.1989	− 0.3400	0.1547	− 0.1852	0.0636	0.1114	0.1333	0.2425	0.0199	− 0.1740	0.2829	1				
Cu	0.5800	0.2786	0.3258	− 0.4368	0.2692	0.4232	0.5442	0.0925	0.1100	− 0.3333	− 0.5571	− 0.3898	0.1725	− 0.2107	1			
Fe	0.0709	− 0.2489	− 0.0546	0.6221	− 0.1287	− 0.1837	0.1001	0.0128	0.0896	− 0.1976	− 0.1117	− 0.1208	− 0.6505	− 0.4577	0.0461	1		
Zn	0.2264	− 0.0678	− 0.0924	0.5816	0.0785	− 0.1280	0.0638	− 0.1068	0.2557	− 0.1546	− 0.0253	− 0.2429	− 0.4986	− 0.5634	− 0.1726	0.5531	1	
Pb	0.3373	0.0240	− 0.0214	0.4651	− 0.0493	− 0.2192	0.2816	0.2776	0.0990	0.0946	− 0.4366	− 0.3521	0.0642	− 0.3392	0.1215	0.1302	0.3411	1

*PO* phenoloxidase, *Px* peroxidase, *PRT* protease, *AntiP* antiprotease, *EST* esterase, *ALP* alkaline phosphatase, *LYZ* lysozyme, *B1* bactericidal activity against *V. anguillarum*, *B2* bactericidal activity against *P. damselae*, *B3* bactericidal activity against *E. coli*, *S* salinity, *T* temperature, *Cd* cadmium, *Cu* copper, *Fe* iron, *Zn* zinc, *Pb* lead

## Discussion

A wide variety of studies have demonstrated that immune functions in bivalves can be disturbed by xenobiotics (Zha et al. 2019; Sun et al. 2020; Tang et al. 2020). Therefore, immune-related biomarkers have been used in pollution biomonitoring studies (Auffret et al. 2006; Cotou et al. 2013; Matozzo et al. 2013), although biochemical biomarkers are currently more widely employed in this type of studies. The carpet shell clam has been proposed as a bioindicator of chemical pollution, and measuring biomarkers in its tissues seems to be a promising approach to monitor the effects of contaminants in the marine environment (Bebianno et al. 2004). In this field study, we measured a set of immunological biomarkers in carpet shell clams collected from three sites in the South Lagoon of Tunis and another site in Louza (RS); monitoring programmes along the Tunisian coast have considered the Louza site as a reference site (Banni et al. 2009). Considering that the temperature and salinity are amongst the abiotic factors which affect biomarker responses in bivalves (Pokhrel et al. 2021), values of physicochemical parameters (salinity, temperature and pH) analysed in seawater from the sampling sites for August and February were used to correlate physicochemical and immune-related parameters. Additionally, concentration data of several metals in the whole soft tissues of clams, measured previously (Mansour et al. 2020), were also integrated.

Regarding immune-related parameters, phenoloxidase activity—which plays a critical role in host defence mechanisms in invertebrates (Muñoz et al. 2006)—decreased in the cell-free haemolymph of clams sampled from the three polluted lagoon compared to the RS for August and February. Similar decreases have also been reported in bivalves exposed to several contaminants (Gagnaire et al. 2004; Bado-Nilles et al. 2009, 2010; Zhou et al. 2010; Díaz-resendiz et al. 2014). For instance, reduced phenoloxidase activity has been reported in the haemocytes of Pacific oysters (*Crassostrea gigas*) exposed to mercury (Gagnaire et al. 2004) and in the cell-free haemolymph of Pacific oysters following in vivo exposure to the soluble fraction of heavy fuel oil (Bado-Nilles et al. 2009) and to the soluble fraction of light crude oil (Bado-Nilles et al. 2010). In another study, phenoloxidase activity was inhibited by Fe^2+^, Mg^2+^, Cu^2+^, Zn^2+^ and Ca^2+^ in the crude fraction of phenoloxidase isolated from abalone *Haliotis discus hannai*, whilst in clam *Scapharca subcrenata*, phenoloxidase activity was stimulated by Mn^2+^, but inhibited by Fe^2+^, Cu^2+^ and Ca^2+^ and Zn^2+^, displaying stimulative effect at 5 mmol L^−1^ (Song et al. 2022). Therefore, these results suggest that phenoloxidase activity could be a highly sensitive immune-related parameter indicating pollutant exposure. Moreover, our results indicated significant seasonality in phenoloxidase activity, which was higher in February than in August (characterised by high seawater temperatures and high salinity values). Similar results have been reported for the scallop (*Chlamys farreri*) during the summer, which is the period corresponding to the reproductive completion of this species and high seawater temperatures (Lin et al. 2012). It is noteworthy that for the Tunisian carpet shell clam population, the reproductive cycle is continuous (Hamida et al. 2004). Thus, the high phenoloxidase activity obtained in the cell-free haemolymph of clams collected in February may be due to high gonadal activity and/or the spawning period between November and December. To the best of our knowledge, few studies have described the relation between phenoloxidase activity and temperature in invertebrates. For instance, Pan et al. (2008) demonstrated a decrease in phenoloxidase activity in the haemocyte lysate supernatant of white shrimp (*Litopenaeus vannamei*) kept at lower temperatures (18 °C and 21 °C) compared to a control group (24 °C). However, the effect of salinity on phenoloxidase activity remains poorly documented in bivalves. Studies carried out on the New Zealand rock oyster (*Saccostrea glomerata*) (Butt et al. 2006) and pearl oyster (*Pinctada imbricata*) (Kuchel et al. 2010) have reported decreased phenoloxidase activity after exposure to low-salinity conditions, whilst a decrease of phenoloxidase activity was recorded in the bivalve *Pinctada fucata* with the increase of salinity (Yang et al. 2022). Thus, these studies and our results demonstrate strong effects of environmental factors on phenoloxidase activity.

Recently, lysozyme activity has been used as a pollution biomarker to monitor the health status of bivalves (Hannam et al. 2010; Luna-Acosta et al. 2010; Matozzo et al. 2012) since several studies have revealed that the presence of contaminants can modulate their activity. In our study, the results pointed to increased lysozyme and esterase activity in the cell-free haemolymph of clams collected from S3 compared to the other sampling sites (including the RS) in February. An in vivo study has reported a decrease in lysozyme activity in the haemolymph of the mussel *Mytilus galloprovincialis* exposed to 1.5 and 15 ng L^−1^ of microplastics, whilst nanoplastic treatments resulted in an increase at 1.5 ng L^−1^ followed by a decrease at 15 and 150 ng L^−1^ (Capolupo et al. 2021). A study carried out on the manila clam (*Ruditapes philippinarum*) has been reported an increase in lysozyme activity during 21 days of exposure to 0.2–20 μg L^−1^ perfluorooctanoic acid (PFOA) (Li et al. 2022). In another study, higher lysozyme activity was observed in the cell-free haemolymph of Manila clams (*R. philippinarum*) collected from a polluted site with high PAH levels in the sediments (Matozzo et al. 2012). A similar pattern was observed in the percentage of esterase-positive cells in the haemocytes of Pacific oysters (*Crassostrea gigas*) exposed to naphthalene (Bado-Nilles et al. 2008), whereas no variations in esterase activity were found in the haemocytes of Pacific oysters after exposure to mercury (Gagnaire et al. 2004) and in the digestive glands of the mussel *M. galloprovincialis* exposed to microplastics (Trestrail et al. 2021). However, several studies have reported inhibited esterase activity in bivalves after exposure to copper (Huang et al. 2018), nano-TiO_2_ (Huang et al. 2016) and PAHs (Wootton et al. 2003; Gagnaire et al. 2006). On the other hand, our results showed a seasonal reduction in lysozyme and esterase activity in the cell-free haemolymph of clams collected in August as compared to February. Esterase activity and lysozyme activity were negatively correlated with temperature and salinity, respectively. These results corroborate the findings of Chu and La Peyre (1989) in the American oyster (*Crassostrea virginica*) where the lysozyme values were higher in winter months than in summer months. Similarly, a reduction of esterase activity was observed in green-lipped (Wang et al. 2011) and thick-shell (Wu et al. 2016) mussels at 30 °C compared to 20 °C and 25 °C, respectively. In contrast, decreases in lysozyme activity have been reported in the blue mussel (*M. galloprovincialis*) (Santarem et al. 1994) and Manila clam (*R. philippinarum*) collected in February rather than in other seasons (Soudant et al. 2004). Another study has reported that lysozyme activity in surf clams (*Mactra veneriformis*) maintained at 10 °C and 30 °C is lower than lysozyme activity in specimens kept at 20 °C (Yu et al. 2009). Our results seem to indicate that the spatial discrimination observed during February suggests immunosuppressive conditions in the S3 polluted area, although this discrimination practically disappeared during August. These results could signify that the low temperatures registered in seawater in February (≈ 13 °C) had a greater influence on lysozyme and esterase activity than did the pollution level in the South Lagoon of Tunis.

Concerning alkaline phosphatase and peroxidase activity, no spatial variations were registered in the cell-free haemolymph of clams sampled in August and February, although some reductions were observed in clams collected in August as compared to February. A previous study has reported an increase in alkaline phosphatase activity in cell-free haemolymph of manila clam (*R. philippinarum*) on days 1, 3, 7 and 10 postexposure to 0.2 μg L^−1^ of PFOA (Li et al. 2022). An in vitro study has reported increases in alkaline phosphatase activity in the cell-free haemolymph of the carpet shell clams (*R. decussatus*) exposed to the combined effects of pyrene and thermal stress (Mansour et al. 2017), and similar variations have been recorded in Indian freshwater mussels (*Lamellidens marginalis*) exposed in vivo to sublethal concentrations of sodium arsenite (Chakraborty et al. 2013). Moreover, increases in alkaline phosphatase activity were recorded in cell-free haemolymph of pearl oyster (*Pinctada maxima*) exposed to thermal stress (Xu et al. 2021). In another study, the highest alkaline phosphatase activity was observed in the cell-free haemolymph of the bivalve *Anomalocardia flexuosa* collected from the polluted site (Carneiro et al. 2021). Furthermore, the authors observed an increase in alkaline phosphatase activity in the animals collected from the relatively non-contaminated site and exposed to thermal stress (Carneiro et al. 2021). For peroxidase, in vitro studies have reported that activity remained unchanged and increased in the haemocytes of Pacific oysters exposed to mercury (Gagnaire et al. 2004) and high concentrations of atrazine (Gagnaire et al. 2005), respectively. In another study, an increment of peroxidase activity was observed in the cell-free haemolymph of the manila clam (*R. philippinarum*) after 21 days of exposure to over an ascending range of concentrations of PFOA (0.2–20 μg L^−1^) (Li et al. 2022). Regarding seasonal and temperature influences, low peroxidase activity levels were registered in Zhikong scallops (*Chlamys farreri*) collected in summer as compared to autumn (Lin et al. 2012), and alkaline phosphatase activity in the cell-free haemolymph of carpet shell clams was lower amongst specimens maintained in vitro at 30 °C than amongst those maintained at 20 °C (Mansour et al. 2017). Overall, our results suggest that both peroxidase activity and alkaline phosphatase activity were inhibited in the cell-free haemolymph of clams collected in August from some sampling sites, which could indicate immunosuppression related to the temperature of the lagoon. Further studies could focus on the effect of the high environmental temperature on such immune activities, with a particular focus on global warming due to climate change, which could be correlated with a poor immune status amongst clams.

Previous studies have described the presence of plasma protease activity in marine bivalve molluscs as a microbicidal mechanism (Muñoz et al. 2003; Allam et al. 2014). However, data on how pollution and temperature affect protease activity in bivalves are scarce. An in vivo study has reported an increase in protease activity in the digestive glands of the mussel *M. galloprovincialis* exposed to high concentrations of microplastics (5 × 10^4^ microplastics L^−1^) (Trestrail et al. 2021). Our observations revealed no spatial variation during August, whilst the higher protease activity was recorded in the cell-free haemolymph of clams collected from S3 compared to clams from S2 in February. Interestingly, comparing the two seasons, an intense reduction in protease activity was reported in the specimens sampled in August compared to February. These results indicate that phenoloxidase, lysozyme, esterase and protease activity in the cell-free haemolymph of clams collected from the polluted sites was influenced by seasonal factors, which is supported by the negative correlations found between protease activity and both salinity and temperature parameters. Regarding antiprotease enzymes, which are involved in the defence of various organisms by regulating and inhibiting the action (Laskowski and Kato 1980), our results showed no variations regardless of sampling location or season. As for bactericidal activity, our results did not reveal variations amongst sampling points for either month (August or February). Thus, bactericidal activity does not appear to provide suitable spatial discrimination for the practical biomonitoring purposes expected from the present work. However, bactericidal activity against *V. anguillarum* and *P. damselae* was reduced in the cell-free haemolymph of clams collected from several sampling points in August as compared to February, which could be related to the general inhibition of most immune-related enzymes tested (phenoloxidase, lysozyme, esterase and protease) in the cell-free haemolymph of clams collected in August from all experimental sampling points.

Principal component analysis was performed to obtain an overall vision of the results on metal concentrations in clam tissue, the abiotic parameters of seawater and immunological biomarkers for all sampling sites and seasons (August and February). Multivariate analysis showed marked seasonal variation characterised by reduced values for immune-related parameters during August, revealing potential immunosuppression under temperature stress. Regarding seasonal variation, the PCA pointed to a clear spatial separation of the sampling sites, with S3 (a chemical industrial area) the most impacted site; the S3 samples had the highest values for lysozyme, esterase and protease activity.

## Conclusions

The present study demonstrates that the immune-related parameters tested in carpet shell clam cell-free haemolymph can be regarded as suitable biomarkers for environmental quality assessments. In fact, the immune-related parameters assayed were modulated by seasonal changes in both environmental and biological factors, which potentially influenced responsiveness and sensitivity to pollutants. The PCA pointed to the S3 as the most impacted site in relation to the immune-related parameters measured. However, the metal analysis revealed that the S1 was the most enriched site in Cd, Fe and Pb. This discrepancy might indicate the presence of other contaminants in the lagoon that could negatively influence immune response regulation. Thus, further studies should be performed to assess the nature and concentration of other pollutants in the studied area and their effects on the immune systems of bivalves. We would like to highlight that the activity of all parameters measured did not always respond simultaneously. Therefore, the present study reinforces that special care must be applied in the identification of confounding factors, such as seasonality, in environmental studies’ designs.

## Data Availability

Not applicable.

## References

[CR1] Allam B, Pales Espinosa E, Tanguy A (2014). Transcriptional changes in Manila clam (*Ruditapes philippinarum*) in response to Brown Ring Disease. Fish Shellfish Immunol.

[CR2] Asokan R, Arumugam M, Mullainadhan P (1997). Activation of prophenoloxidase in the plasma and haemocytes of the marine mussel *Perna viridis* Linnaeus. Dev Comp Immunol.

[CR3] Auffret M, Rousseau S, Boutet I (2006). A multiparametric approach for monitoring immunotoxic responses in mussels from contaminated sites in Western Mediterranea. Ecotoxicol Environ Saf.

[CR4] Bado-Nilles A, Gagnaire B, Thomas-Guyon H (2008). Effects of 16 pure hydrocarbons and two oils on haemocyte and haemolymphatic parameters in the Pacific oyster, *Crassostrea gigas* (Thunberg). Toxicol Vitr.

[CR5] Bado-Nilles A, Quentel C, Auffret M (2009). Immune effects of HFO on European sea bass, *Dicentrarchus labrax*, and Pacific oyster, *Crassostrea gigas*. Ecotoxicol Environ Saf.

[CR6] Bado-Nilles A, Renault T, Faury N (2010). *In vivo* effects of LCO soluble fraction on immune-related functions and gene transcription in the Pacific oyster, *Crassostrea gigas* (Thunberg). Aquat Toxicol.

[CR7] Bahi A, Guardiola FA, Messina C (2017). Effects of dietary administration of fenugreek seeds, alone or in combination with probiotics, on growth performance parameters, humoral immune response and gene expression of gilthead seabream (*Sparus aurata* L.). Fish Shellfish Immunol.

[CR8] Banni M, Bouraoui Z, Ghedira J (2009). Seasonal variation of oxidative stress biomarkers in clams *Ruditapes decussatus* sampled from Tunisian coastal areas. Environ Monit Assess.

[CR9] Bebianno MJ, Géret F, Hoarau P (2004). Biomarkers in *Ruditapes decussatus*: a potential bioindicator species. Biomarkers.

[CR10] Bejaoui S, Rabeh I, Telahigue K, et al (2020) Assessment of oxidative stress, genotoxicity and histopathological responses in the digestive gland of *Ruditapes decussatus* collected from northern Tunisian lagoons. Sci Mar 84:403–420. 10.3989/scimar.05054.23A

[CR11] Bejaoui S, Telahigue K, Chetoui I (2018). Integrated effect of metal accumulation, oxidative stress responses and DNA damage in *Venerupis decussata* gills collected from two coast Tunisian lagoons. J Chem Environ Biol Eng.

[CR12] Bradford M (1976). A rapid and sensitive method for quantification of microgram quantities of protein using the principle of protein dye binding. Anal Biochem.

[CR13] Breitwieser M, Viricel A, Graber M (2016). Short-term and long-term biological effects of chronic chemical contamination on natural populations of a marine bivalve. PLoS ONE.

[CR14] Butt D, Shaddick K, Raftos D (2006). The effect of low salinity on phenoloxidase activity in the Sydney rock oyster, *Saccostrea glomerata*. Aquaculture.

[CR15] Capó X, Tejada S, Box A (2015). Oxidative status assessment of the endemic bivalve *Pinna nobilis* affected by the oil spill from the sinking of the Don Pedro. Mar Environ Res.

[CR16] Capolupo M, Valbonesi P, Fabbri E (2021). A comparative assessment of the chronic effects of micro-and nano-plastics on the physiology of the mediterranean mussel *Mytilus galloprovincialis*. Nanomaterials.

[CR17] Carneiro AP, Soares CHL, Pagliosa PR (2021). Does the environmental condition affect the tolerance of the bivalve *Anomalocardia flexuosa* to different intensities and durations of marine heatwaves?. Mar Pollut Bull.

[CR18] Chakraborty S, Ray M, Ray S (2013). Cell to organ: physiological, immunotoxic and oxidative stress responses of *Lamellidens marginalis* to inorganic arsenite. Ecotoxicol Environ Saf.

[CR19] Chalghmi H, Bourdineaud JP, Chbani I (2020). Occurrence, sources and effects of polycyclic aromatic hydrocarbons in the Tunis lagoon, Tunisia: an integrated approach using multi-level biological responses in *Ruditapes decussatus*. Environ Sci Pollut Res.

[CR20] Chalghmi H, Bourdineaud JP, Haouas Z (2016). Transcriptomic, biochemical, and histopathological responses of the clam *Ruditapes decussatus* from a metal-contaminated Tunis Lagoon. Arch Environ Contam Toxicol.

[CR21] Chu FLE, La Peyre JF (1989). Effect of environmental factors and parasitism on hemolymph lysozyme and protein of American oysters (*Crassostrea virginica*). J Invertebr Pathol.

[CR22] Cotou E, Tsangaris C, Henry M (2013). Comparative study of biochemical and immunological biomarkers in three marine bivalves exposed at a polluted site. Environ Sci Pollut Res.

[CR23] Cravo A, Pereira C, Gomes T (2012). A multibiomarker approach in the clam *Ruditapes decussatus* to assess the impact of pollution in the Ria Formosa lagoon, South Coast of Portugal. Mar Environ Res.

[CR24] Díaz-resendiz KJG, Romero-bañuelos CA, Robledo-marenco ML (2014). Deregulation of the humoral immune response of the oyster (*Crassostrea corteziensis*) exposed to naphthalene. Invertebr Surviv J.

[CR25] Gagnaire B, Renault T, Bouilly K (2005). Study of atrazine effects on Pacific oyster, *Crassostrea gigas*, haemocytes. Curr Pharm Des.

[CR26] Gagnaire B, Thomas-Guyon H, Burgeot T, Renault T (2006). Pollutant effects on Pacific oyster, *Crassostrea gigas* (Thunberg), hemocytes: screening of 23 molecules using flow cytometry. Cell Biol Toxicol.

[CR27] Gagnaire B, Thomas-Guyon H, Renault T (2004). *In vitro* effects of cadmium and mercury on Pacific oyster, *Crassostrea gigas* (Thunberg), haemocytes. Fish Shellfish Immunol.

[CR28] Gola D, Kumar Tyagi P, Arya A (2021). The impact of microplastics on marine environment: a review. Environ Nanotechnol Monit Manag.

[CR29] Guardiola FA, Bahi A, Esteban MA (2018). Effects of dietary administration of fenugreek seeds on metabolic parameters and immune status of gilthead seabream (*Sparus aurata* L.). Fish Shellfish Immunol.

[CR30] Guardiola FA, Cuesta A, Arizcun M (2014). Comparative skin mucus and serum humoral defence mechanisms in the teleost gilthead seabream (*Sparus aurata*). Fish Shellfish Immunol.

[CR31] Hamida L, Medhiouband MN, Cochard JC, et al (2004) Étude comparative du cycle de reproduction de la palourde *Ruditapes decussatus* en milieu naturel (sud Tunisie) et contrôlé (écloserie). Cah Biol Mar 45:291–303

[CR32] Hanif A, Bakopoulos V, Dimitriadis GJ (2004). Maternal transfer of humoral specific and non-specific immune parameters to sea bream (*Sparus aurata*) larvae. Fish Shellfish Immunol.

[CR33] Hannam ML, Bamber SD, Galloway TS (2010). Effects of the model PAH phenanthrene on immune function and oxidative stress in the haemolymph of the temperate scallop *Pecten maximus*. Chemosphere.

[CR34] Hong WJ, Jia H, Li YF (2016). Polycyclic aromatic hydrocarbons (PAHs) and alkylated PAHs in the coastal seawater, surface sediment and oyster from Dalian, Northeast China. Ecotoxicol Environ Saf.

[CR35] Huang X, Jiang X, Sun M (2018). Effects of copper on hemocyte parameters in the estuarine oyster *Crassostrea rivularis* under low pH conditions. Aquat Toxicol.

[CR36] Huang X, Lin D, Ning K (2016). Hemocyte responses of the thick shell mussel *Mytilus coruscus* exposed to nano-TiO_2_ and seawater acidification. Aquat Toxicol.

[CR37] Jouini Z, Ben CR, Moussa M (2005). Caractéristiques du Lac Sud de Tunis après sa restauration Characteristics of the South Lake of Tunis after restoration. Mar Life.

[CR38] Kuchel RP, Raftos DA, Nair S (2010). Immunosuppressive effects of environmental stressors on immunological function in *Pinctada imbricata*. Fish Shellfish Immunol.

[CR39] Laskowski M, Kato I (1980). Protein inhibitors of Proteinases. Annu Rev Biochem.

[CR40] Li F, Liu Z, Yao L (2022). Immunotoxicity of perfluorooctanoic acid to the marine bivalve species *Ruditapes philippinarum*. Environ Toxicol Chem.

[CR41] Lin T, Zhou K, Lai Q (2012). Seasonal variations of water temperature, food availability, size, and reproduction on the hemocyte parameters in the scallop *Chlamys farreri*. J Shellfish Res.

[CR42] Luna-Acosta A, Bustamante P, Godefroy J (2010). Seasonal variation of pollution biomarkers to assess the impact on the health status of juvenile Pacific oysters *Crassostrea gigas* exposed *in situ*. Environ Sci Pollut Res.

[CR43] Mansour C, Ben Taheur F, Mzoughi R, Mosbahi D (2021) Hydrocarbon levels and biochemical biomarkers in the clam *Ruditapes decussatus* collected from Tunis lagoon (Tunisia), in Proceedings of the MOL2NET'21, Conference on Molecular, Biomedical & Computational Sciences and Engineering, 7th ed., 25 January 2021–30 January 2022, MDPI: Basel, Switzerland. 10.3390/mol2net-07-12043

[CR44] Mansour C, Guardiola FA, Esteban MÁ, Mosbahi DS (2017). Combination of polycyclic aromatic hydrocarbons and temperature exposure: *In vitro* effects on immune response of European clam (*Ruditapes decussatus*). Fish Shellfish Immunol.

[CR45] Mansour C, Guibbolini M, Rouane Hacene O (2020). Oxidative stress and damage biomarkers in clam *Ruditapes decussatus* exposed to a polluted site: the reliable biomonitoring tools in hot and cold seasons. Arch Environ Contam Toxicol.

[CR46] Matozzo V, Binelli A, Parolini M (2012). Biomarker responses in the clam *Ruditapes philippinarum* and contamination levels in sediments from seaward and landward sites in the Lagoon of Venice. Ecol Indic.

[CR47] Matozzo V, Giacomazzo M, Finos L (2013). Can ecological history influence immunomarker responses and antioxidant enzyme activities in bivalves that have been experimentally exposed to contaminants? A new subject for discussion in “eco-immunology” studies. Fish Shellfish Immunol.

[CR48] Moncaleano-Niño AM, Gómez-Cubillos MC, Luna-Acosta A (2022). Monitoring metallothionein-like protein concentrations and cholinesterase activity in tropical cup oysters as biomarkers of exposure to metals and pesticides in the southern Caribbean, Colombia. Environ Sci Pollut Res.

[CR49] Monserrat JM, Geracitano LA, Bianchini A (2003). Current and future perspectives using biomarkers to assess pollution in aquatic ecosystems. Comments Toxicol.

[CR50] Muñoz P, Meseguer J, Esteban MÁ (2006). Phenoloxidase activity in three commercial bivalve species. Changes due to natural infestation with *Perkinsus atlanticus*. Fish Shellfish Immunol.

[CR51] Muñoz P, Vance K, Gómez-Chiarri M (2003). Protease activity in the plasma of American oysters, *Crassostrea virginica*, experimentally infected with the protozoan parasite *Perkinsus marinus*. J Parasitol.

[CR52] Mzoughi N, Chouba L (2011). Distribution of trace metals, aliphatic hydrocarbons and polycyclic aromatic hydrocarbons in sediment cores from the Sicily Channel and the Gulf of Tunis (south-western Mediterranean Sea). Environ Technol.

[CR53] Pan LQ, Hu FW, Jing FT, Liu HJ (2008). The effect of different acclimation temperatures on the prophenoloxidase system and other defence parameters in *Litopenaeus vannamei*. Fish Shellfish Immunol.

[CR54] Parry RM, Chandan RC, Shahani KM (1965). A rapid and sensitive assay of muramidase. Proc Soc Exp Biol Med.

[CR55] Pokhrel P, Suzuki J, Akther S, Fujita M (2021). Physiological and biochemical responses of brackish-water clam *Corbicula japonica* under global-warming conditions: water temperature, salinity, and food availability. Ecol Indic.

[CR56] Quade MJ, Roth JA (1997). A rapid, direct assay to measure degranulation of bovine neutrophil primary granules. Vet Immunol Immunopathol.

[CR57] R Core Team (2020) R: a language and environment for statistical computing. R Foundation for Statistical Computing, Vienna, Austria. https://www.R-project.org/. Accessed 26 Apr 2020

[CR58] Sandrini-Neto L, Pereira L, Martins CC (2016). Antioxidant responses in estuarine invertebrates exposed to repeated oil spills: effects of frequency and dosage in a field manipulative experiment. Aquat Toxicol.

[CR59] Santarem MM, Robledo JAF, Figueras A (1994). Seasonal changes in hemocytes and serum defense factors in the blue mussel *Mytilus galloprovincialis*. Dis Aquat Organ.

[CR60] Shah SB (2021) Heavy metals in the marine environment—an overview. In: Heavy Metals in Scleractinian Corals. Springer Briefs in Earth Sciences. Springer, Cham. 1–26. 10.1007/978-3-030-73613-2_1

[CR61] Solé M (2000). Assessment of the results of chemical analyses combined with the biological effects of organic pollution on mussels. TrAC - Trends Anal Chem.

[CR62] Song X, Jiang J, Xing J, Zhan W (2022). Isolation and biochemical characteristics analyses of phenoloxidases (POs) in three cultured mollusk species. J Ocean Univ China.

[CR63] Soudant P, Paillard C, Choquet G (2004). Impact of season and rearing site on the physiological and immunological parameters of the Manila clam *Venerupis* (=*Tapes*, =*Ruditapes*) *philippinarum*. Aquaculture.

[CR64] Sun S, Shi W, Tang Y (2020). Immunotoxicity of petroleum hydrocarbons and microplastics alone or in combination to a bivalve species: synergic impacts and potential toxication mechanisms. Sci Total Environ.

[CR65] Sunyer JO, Tort L (1995). Natural hemolytic and bactericidal activities of sea bream *Sparus aurata* serum are effected by the alternative complement pathway. Vet Immunol Immunopathol.

[CR66] Tang Y, Rong J, Guan X (2020). Immunotoxicity of microplastics and two persistent organic pollutants alone or in combination to a bivalve species. Environ Pollut.

[CR67] Trestrail C, Walpitagama M, Miranda A (2021). Microplastics alter digestive enzyme activities in the marine bivalve *Mytilus galloprovincialis*. Sci Total Environ.

[CR68] Wang Y, Hu M, Shin PKS, Cheung SG (2011). Immune responses to combined effect of hypoxia and high temperature in the green-lipped mussel *Perna viridis*. Mar Pollut Bull.

[CR69] Wootton EC, Dyrynda EA, Pipe RK, Ratcliffe NA (2003). Comparisons of PAH-induced immunomodulation in three bivalve molluscs. Aquat Toxicol.

[CR70] Wu F, Lu W, Shang Y (2016). Combined effects of seawater acidification and high temperature on hemocyte parameters in the thick shell mussel *Mytilus coruscus*. Fish Shellfish Immunol.

[CR71] Xu Y, Zhang Y, Liang J (2021). Impacts of marine heatwaves on pearl oysters are alleviated following repeated exposure. Mar Pollut Bull.

[CR72] Yang J, Yang J, Chen M (2022). Physical responses of *Pinctada fucata* to salinity stress. Front Mar Sci.

[CR73] Yap CK, Sharifinia M, Cheng WH (2021). A commentary on the use of bivalve mollusks in monitoring metal pollution levels. Int J Environ Res Public Health.

[CR74] Yu JH, Song JH, Choi MC, Park SW (2009). Effects of water temperature change on immune function in surf clams, *Mactra veneriformis* (Bivalvia: Mactridae). J Invertebr Pathol.

[CR75] Zaynab M, Fatima M, Sharif Y (2021). Health and environmental effects of silent killers Organochlorine pesticides and polychlorinated biphenyl. J King Saud Univ - Sci.

[CR76] Zha S, Rong J, Guan X (2019). Immunotoxicity of four nanoparticles to a marine bivalve species, *Tegillarca granosa*. J Hazard Mater.

[CR77] Zhang X, Li D, Wang X (2021). Exploration of polycyclic aromatic hydrocarbon distribution in the sediments of marine environment by hydrodynamic simulation model. Mar Pollut Bull.

[CR78] Zhou J, Cai Z, hua, Zhu X shan,  (2010). Innate immune parameters and haemolymph protein expression profile to evaluate the immunotoxicity of tributyltin on abalone (*Haliotis diversicolor supertexta*). Dev Comp Immunol.

